# The Scientific Method in Veterinary Education

**Published:** 1884-01

**Authors:** 


### EDITORIAL DEPARTMENT.

### THE SCIENTIFIC METHOD IN VETERINARY EDUCATION.

IT is the duty of professional men to take an active interest in the future of the profession to which they belong. Do they do this in this country? Do we find that scientific spirit permeating our professional societies, or associations, which is so common among the same organizations in Europe? Emphatically no! The majority of American professionals—we are speaking of the medical profession—are too much infected with that worldly disease—'money getting'—to take any very active interest in professional matters. "Sufficient unto the day is the evil thereof" is the professional motto. "I am doing well enough, what do I care about this research business," the too frequent remark. The fault lies deeper than the profession. It existed before technical schools were ever thought of here. It retards the progress of human and is bound to be the bane of Veterinary Medicine. We inherited it from the, so called, 'mother country.' It is an English failing; that is, it is common to every country where the English language is spoken. This great failure is, the utter want of a true scientific, that is, spirit of research permeating the profession as a body; finding active supporters in a large number of representative men in the profession; recognized as one of the great cardinal necessities, and as such promoted and supported by government. Shall we ever attain this end? When one looks at the results in England we have every reason to despair, for the British government has never done anything to support original research in the field of medicine. In fact, the spirit of that nation (which is one of sentimental gush, not reason) is opposed to every advance in the study of disease or its causes as is evinced by its action on the vivisection question. (While in Germany I made the acquaintance of a very clever and noted English doctor, who had come there to make some special studies for which experiments on animals were necessary; he assured me that he had to get away on the false pretense 'of ill health,' and that should it be known at home, that he was engaged in such work, it would almost ruin his practice. The results of his studies have since been published, as from a German author, and have been of great *practical* benefit to humanity. Sentiment, unless controlled by cold blooded reason is worse than useless.)

Britain has had her Bacons, Newtons, Darwins and Huxleys but they have not been representative men in any sense of the word. They represented no scientific class; no scientific spirit which they shared in common with any body of their fellow citizens. They stand out as oases of intellectual light; like the electric lights in our cities they serve only to make still more hideous the intellectual death and darkness by which they were surrounded. They owed their greatness to the great natural gifts of which they were posessed, and to the fact that they were born under such favorable circumstances that they could indulge their scientific tastes as a pleasure. In reality when one makes a careful review of English or American life such men appear so phenominal that we might be justified in classing them among the "natural curiosites." The abnormal unfortunates displayed in our "Dime museums" are by no means so rare as men engaged in original research in the scientific professions of these two countries. Yet we are not without the material, and an abundance of it, out of which such men are developed. The reason that they do not come to maturity is that we smother them in their youth. The profession has no place for them, they become uncomfortable members in our societies, their colleagues seek to stamp them out as they would a dangerous pest, or to muzzle them as they would a vicious dog. Do-nothingism is the characteristic of most of our medical societies. A respectable regard for, and repetition of the work of the ancient and bygone is their chief aim. We have said that this want of support of scientific research is a national fault. Original research can never succeed as the result of private endeavour. The possession of the means to proceed with, and the brain to conduct such work is not frequently found in one and the same person. Advancement in this field depends upon a great number of workers, and a great divergence in minds and methods.

Were I asked to state in a few words our politico-scientific creed it would be as follows: "politically republican, (but not of the party) scientifically, monarchical."

The governments of the United States and Britain are essentially of the people though that of the latter has a monarchical figure head. Public interests are dependent upon the will of the people. Without the popular support, measures are seldom adopted or vetoed.

The reason that scientific research does not find popular support and that means governmental, in these countries, is to be sought first, in the want of that education of the masses which is necessary to its appreciation, and second, in the great amount of freedom from any general responsibility which the individual feels in a republic. So long as the individual is well and prospering he cares little for national, or special interest. Preservation of life seldom troubles the individual until he feels it endangered. Nothing but the eruption of the most dangerous pests ever awakens the public mind to the necessity of studying into their nature and causes. With pests as with war the family hearth must be endangered before the average republican citizen finds interest in the public welfare engrossing his mind.

In monarchical governments the contrary is the case. Here we find that the interests of each and every individual become in one way or another those of a single person. Hence the the term 'Parental Governments.' It is a form of selfishness perhaps, yet whatever affects the nation must react upon its sovereign, or the ruling classes. No matter how bad his private character, the monarch cannot afford to ignore the interests of the people whether of a hygienic or monetary sort. National prosperity means peace of mind and prosperity to the monarch, while national adversity, of whatever kind, brings its sequential influence. The death of hundreds of thousands of people from the cholera would hardly affect the stability of our government, yet if the disease should extend over Germany alone to such extent it would soon be followed by French aggression, and the consequent loss of life and teritory, if not the destruction of the German Empire which would all exert a very disastrous influence upon the ruling family.

One who has not read of them can scarcely realize the amount of misery, even to national weakness, that has been produced by ravaging animal pests, especially the Rinderpest in past centuries. Nations have been crippled, aye almost ruined by them. An old fashioned invasion of the cattle-plague into Germany would more than settle the question of the balance of power between France and Germany for a long time without their ever having to call upon their armies. In ancient days the fate of nations has depended upon the number and condition of their cavalry.

It is thus easily seen that matters affecting the people of a nation are far more likely to receive the recognition of an individual monarch, or a small ruling class like the nobility of Europe, than either the citizen, or the representatives of a republican country.

Hence it is, that these monarchs are obliged to study the welfare of the people, for that which affects the people must affect the ruler.

To develope the resources of the country is more to the rulers interest than to the individual citizen, or, any one class of people. To keep the people well, contented and happy, is a matter of good policy, if nothing more.

This cannot be done without the aid of scientists; hence it is that we see Science flourishing amidst the black terrorism of Russia and the stern despotism of Germany. Sunny France, through a republic, is the honorable exception among republics, for she has not thrown off the good in her monarchical inheritances, and is second to no country in her liberal support of Science, and her disciples—The rulers of these countries truly realize that "*Scientia est potentia*!"—Knowledge is power!

Comparison between any uncivilized nation with a civilized is all that is necessary to prove this. It is the one powerful factor which has raised the American of to day superior to the Indian. Science is the conqueror of the world. Science is the sole producer of wealth, comfort, and prosperity. Science is the only power able to fight with disease, or cope with death. Everything we possess has been given to us by the original researchers of the world.

The prosperity of nations has been and is dependent upon the results of scientific work, and yet America does nothing to support it. What would be the value of all the wealth in this country, of our mills and foundries, could the results of scientific research be at once taken from mens minds and drop out of existence and all research cease forever?

What is the value of all this wealth in comparison to health. The sickly millionaire would willingly divide his wealth with the poorest laborer, when his life is threatened by some infectious disease. The world would stand still, the ravaging pest, animal as well as human, would still be over-running the earth—death, misery, and devastation following in their path were it not for the researches of scientists, past and present.

If it is so necessary for monarchs to thus support original research, is it any the less necessary that it should find favor and support in republics. Is it not really a national disgrace that original research finds no home in this country? Where money can be made, wealthy men or corporations, are willing to find the means for applying the results obtained by scientists in their laboratories. But of what nationality are these scientists? Are they Americans? They are generally Europeans; by far the greater number of them Germans.

It is said that the writer is 'German mad,' so that my testimony may not be of much value. Let us see what an Englishman has to say, remembering that every word he writes is doubly applicable to this country.

Prof. E. Ray Lankester, in his address on Biology, delivered before the British Association for the Advancement of Science, (Sept. 21st, 1883) says:

"The knowledge of the growth of the chick from the egg and of other organisms from similarly constituted beginnings has been slowly and continuously gained by prodigious labor, extending over gereration after generation of students who have occupied the laboratories and lived on the stipends provided by the governments of European States—not English, but chiefly German. It is this history of the development of the individual animal and plant from a simple homogeneous begining to a complex heterogeneous adult which has furnished a starting-point for the wide-reaching doctrine of evolution. It is this knowledge, coupled with the knowledge of the myriad details of structure of all kinds of animals and plants which the faithful occupants of laboratories and the guardians of biological collections have in the past one hundred [years laboriously searched out and recorded—it is this which enabled Darwin to propound, to test, and to firmly establish his theory of the origin of species by natural selection, and finally to bring the origin, development, and progress of man also into the arena of physical science."

"It may be laid down as a general proposition that scientific discovery has only been made by one of two classes of men, namely: 1. Those whose time could be devoted to it in virtue of their possessing inherited fortunes; 2. Those whose time could be devoted to it in virtue of their possessing a stipend or endowment especially assigned to them for that purpose. Those who wish to defend the present neglect of the Government, and of public institutions, to provide means for the carrying on of scientific research in this country, are accustomed to declare as a justification for this neglect that we do very well without such provision, inasmuch as the cultivation of science here flourishes in the hands of those who are in a position of pecuniary independence. The reply to this is obvious. If those few of our countrymen who, by accident, are placed in an independent position, show such ability in the prosecution of scientific research, how much more would be effected in the same direction were the machinery provided to enable those also who are not accidentally favored by fortune, to enter upon the same kind of work?"

"It is altogether a mistake to suppose that the existence among us of a few very eminent men is any evidence that we are contributing largely to the hard work of careful study and observation, which really forms the material upon which the conclusions of eminent discoverers are based. In every department of biological knowledge the hard work of investigation is being carried on by the well-trained army of German observers. Whether you ask the zoölogist, the botanist, the physiologist, or the anthropologist, you will get the same answer. It is to German sources that he looks for new information; it is in German workshops that discoveries, each small in itself, but gradually leading up to great conclusions, are daily being made. To a very large extent the business of those who are occupied with teaching or applying biological science, in this country, consists in making known what has been done in German laboratories. Our English students flock to Germany to learn the methods of scientific research; and to such a state of weakness is English science reduced for want of proper nurture and support, that even on some of the rare occasions when a capable investigator of biological problems has been required for the public service, it has been necessary to obtain the assistance of a foreigner, trained in the laboratories of Germany."

"In the German Empire, with a population of 45,000,000, there are twenty-one universities. These universities are very different from anything which goes by the name in this country. Among its other arrangements devoted to the study and teaching of all branches of learning and science, each university has five institutes or establishments devoted to the prosecution of researches in biological science. These are respectively the physiological, the zoölogical, the anatomical, the pathological, and the botanical. In one of these universities of average size each of the institutes named consists of a spacious building containing many rooms fitted as workshops, provided with instruments, a museum, and, in the last instance, with an experimental garden. All this is provided and maintained by the State."

It is just this which I have been vainly trying to bring about in this country with reference to the establishment of Veterinary Schools. Veterinary Science, as we understand it, is not the Veterinary medicine taught in the few schools at present existing. It is not the 'Horse-doctor' knowledge which the people think sufficient for the Veterinary practitioner. The Veterinary Science which we have in mind would serve as the very foundation stone for further progress in human medicine, as well as a supporter of it in every department. A Scientific Veterinary School such as this country *must have*; such as it *will have* some day (when some wealthy Americans become inpressed with the idea that there is nothing the country so much needs save a medical school of the same sort) must be organized something in this manner.

Its purposes should be:

1. To study the results of disease in all domestic animals to their minutest details; to compare them with each other, and with the results of similar diseases in man.

2. To study the relation of the diseases of our domestic animals to the human being; as well as to trace the connection between human diseases and the food-products of our animals, such as flesh, milk, etc.

3. To study the causes of all diseases of animals by most careful observations and experiments, including examinations of the earth, air, water, feed, transport, or anything which can exert any influence in the generation or spread of diseases.

4. To endeavor to discover means by which the action of the causes of disease may be prevented, or their generation interfered with.

5. To make careful studies of the actions of drugs upon animals, and also to improve and enlarge upon the operations of Veterinary Surgery.

6. To make careful and scientific studies of the practical value of the different kinds of animal foods:

1. As to their affect on the general health:
2. As to their affect and the average quantity necessary to produce a given quantity of work.
3. As to their affects upon animals which either in the form of flesh, or through their products, milk, are used as human food.

7. To apply the result of all this research in a *practical, yet scientific manner to the education of students*, so that they may become not only *very practical men*, but be so filled with *this scientific spirit*, that they may be accurate students and observers, and thus while engaged in practice be able to add their mites to the treasury of knowledge, and be useful citizens to the State: in fact, we must produce something else than *routine practitioners;* which would be nothing; empiricism can do that.

